# Food Trucks: Assessment of an Evaluation Instrument Designed for the Prevention of Foodborne Diseases

**DOI:** 10.3390/nu11020430

**Published:** 2019-02-19

**Authors:** Lígia Isoni Auad, Verônica Cortez Ginani, Eliana dos Santos Leandro, Elke Stedefeldt, Sascha Habu, Eduardo Yoshio Nakano, Aline Costa Santos Nunes, Renata Puppin Zandonadi

**Affiliations:** 1 Department of Nutrition, Faculty of Health Sciences, University of Brasilia (UnB), Campus Darcy Ribeiro, Asa Norte, Brasilia DF 70910-900, Brazil; vcginani@gmail.com (V.C.G.); elisanleandro@yahoo.com.br (E.d.S.L.); renatapz@yahoo.com.br (R.P.Z.); 2 GeQual—Study Group of Food Quality, Centro de Desenvolvimento do Ensino Superior em Saúde, Universidade Federal de São Paulo, São Paulo SP 04021-001, Brazil; elkesnutri@gmail.com; 3 Department of Food Technology, Universidade Tecnológica Federal do Paraná (UTFPR), Campus Medianeira, Medianeira PR 85884-000, Brazil; sashabu@yahoo.com.br; 4 Department of Statistics, Institute of Exact Sciences, University of Brasilia (UnB), Campus Darcy Ribeiro, Asa Norte, Brasilia DF 70910-900, Brazil; eynakano@gmail.com; 5 Department of Pharmacy, Faculty of Health Sciences, University of Brasília (UnB), Campus Darcy Ribeiro, Asa Norte, Brasilia DF 70910-900, Brazil; alinecostasn@gmail.com

**Keywords:** food truck, food safety, foodborne diseases, risk assessment

## Abstract

This study aimed to validate an evaluation instrument for food trucks (FTs) regarding its internal consistency and to establish a score classification according to the food contamination probability assessment. The instrument was applied in 44 food trucks (convenience sample), along with microbial analysis, in the Federal District, Brazil. After its application, sample collection and statistical analysis, the evaluation instrument was reduced to a 22-item final version. FTs were divided into three groups according to their ready-to-eat foods. Food trucks from Group A (hot and cold sandwiches) presented the highest percentage of contamination. The lowest percentage of contamination was observed in food trucks from Group C (regional and international cuisine). The application of the validated evaluation instrument to the 44 food trucks revealed that none achieved 100% adequacy. The reproducibility analysis exhibited an Intraclass Correlation Coefficient (ICC) value of 0.780 (CI 95%: 0.597; 0.880), indicating good reproducibility of the instrument. The reliability assessment presented a Kuder–Richardson Formula 20 (KR-20) value of 0.627 and a Cronbach’s alpha coefficient of 0.634, indicating good internal consistency. The proposed classification score was obtained by assigning 1 point for each item with an inadequate response, and the final score may vary between 0 and 20 points. Food trucks with up to 11 points exhibit low probability of contamination and low risk of foodborne diseases, while food trucks scoring 12 or more points exhibit a high probability of contamination and high risk of foodborne diseases. The evaluation instrument will allow effective assessment of the hygienic–sanitary practices and conditions of food trucks and potentially ensure consumers’ access to safe food.

## 1. Introduction

The feeding profile of the population has been changing due to the increasing demand for food diversification, availability, and accessibility, along with the modern lifestyle, which is characterized by a lack of time for food preparation and consumption. It is noteworthy that people are eating out and the meals prepared at home have been replaced by fast meals, in places of easy access and with fast service, like food trucks (FTs) [1]. The FT industry has been booming in both developed and developing countries, spurred by the process of globalization and urbanization, and is now one of the best performing segments in the foodservice.

FTs are itinerant miniature commercial kitchens, which usually use commissaries for food storage and preparation. Due to the high number of ingredients and processes, as well as transportation to the selling points, FTs require special attention to time and temperature control during the food process chain, what brings them closer to brick and mortar establishments. On the other hand, FTs are closely linked to the street food vending sector if the selling points and the exposure to the environmental conditions are considered [2]. 

FT activity generally escapes from effective food safety regulation and inspection [3]. A study performed in California by Faw and Tuttle [4] revealed that 94.73% of the FTs assessed exhibited at least one critical risk factor that contributes to foodborne diseases (FBDs). This research also indicated that mobile FTs demonstrate attributes comparable to fixed food facilities and therefore, would benefit from similar inspection practices. The factors that can cause FBD outbreaks in Brazil and in other countries are time and temperature aspects; contamination by food handlers, equipment and utensils; contaminated water and raw material; and indirect contamination [5]. These factors reflect complex and interrelated situations rather than isolated hazards and represent situations where FBD can be avoided by risk mediation according to the context of its occurrence [6]. 

FBDs remain a public health challenge despite the constant global effort from industries and governments to ensure the hygienic–sanitary quality of food production. According to the World Health Organization (WHO) ‘Estimates of the Global Burden of Foodborne Diseases’ [7], approximately 600 million cases of illness and 420,000 deaths in 2010 were caused by 31 foodborne hazards, including bacteria, viruses, parasites, toxins and chemicals. In Brazil, the National Notifiable Diseases Information System (Sistema de Informação de Agravos de Notificação—SINAN) notified a total of 6632 FBD outbreaks from 2007 to 2016, among 469,482 people who were exposed to the hazards during the same period [8].

The development and adoption of intervention strategies can play a key role in the prevention and control of foodborne diseases in FTs. However, there is no record in the scientific literature of an investigation of possible strategies to prevent contamination in these vehicles. Instruments for the verification of non-conformities in loco that are related to the occurrence of poor hygiene and handling practices may represent an interesting approach to control the production process and provide safe food. Due to its low cost, high applicability and accessible design, a checklist instrument represents an attractive alternative to conducting inspections in FTs by health surveillance auditors and for the decision-making process of FT vendors willing to comprehend and implement proper food safety practices. It can also be used as a diagnostic tool for the assessment of the hygienic–sanitary practices and conditions in these types of food operations.

In Brazil, Auad et al. [9] developed an evaluation instrument for the assessment of the hygiene practices and conditions of FTs. This checklist was developed based on the Brazilian good manufacturing practices regulation and the General Principles of Food Hygiene of the Codex Alimentarius [10], focusing on the most significant factors to be controlled to prevent FBD. This checklist was also validated, considering content and semantics, by the Delphi method and, therefore, may represent a scientifically founded alternative to assist the implementation of a FBD prevention system.

In the current study, the instrument previously designed and validated regarding the importance (content validation) and clarity (semantic evaluation) of their items by the Delphi technique [9] was used to assess Brazilian FTs’ practices and conditions. Its application, which is described in this study, enabled a shorter version of an original evaluation instrument. Given the absence of studies concerning the prevention of foodborne diseases in FTs and of validated instruments available for assessing the hygienic–sanitary practices and conditions of FTs, the objectives of this study were to measure internal consistency and to establish an evaluation score for the checklist according to the food contamination probability assessment.

## 2. Materials and Methods 

This cross-sectional study was conducted in the Federal District, Brazil, from December 2017 to April 2018, with 44 FTs that were randomly selected because the location and frequency of FT activity is not standardized or previously informed. The exclusion criterion was the FT not willing to participate in the research.

The ethical and methodological aspects of this study had approval by the Ethics Commission of the University of Brasília (UnB), registered under number CEP no. 2.178.214. We assured anonymity and confidentiality of participants throughout the study.

**Figure 1:**
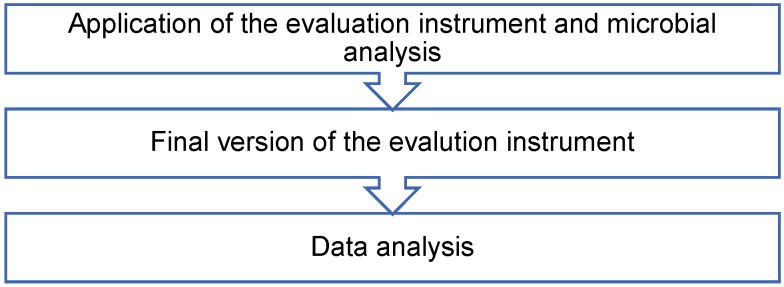
Layout of the study methodology.

This study was carried out in three phases. Figure 1 shows the schematic methodology outline. 

### 2.1. Stage 1—Application of the Evaluation Instrument and Microbial Analysis

A checklist instrument was chosen to evaluate the hygienic–sanitary conditions and practices of FTs. This evaluation method was chosen due to its low cost, high applicability, and accessible design [11]. The technical and legal basis of the evaluation instrument were the Brazilian Ministry of Health Anvisa (Agência Nacional de Vigilância Sanitária—National Health Surveillance Agency) resolutions No. 216 [12] and No. 275 [13]—which are based on the the General Principles of Food Hygiene of the Codex Alimentarius [10]—and the local Brazilian legislations of the Federal District, Normative Instruction 11 (IN 11) [14] and legislation No. 5.627 [15].

After the development of the initial draft, composed of 29 items, the evaluation instrument was subjected to the validation process, which considered the content validation and semantic evaluation simultaneously. The validation process was performed using a two-round Delphi approach, as described by Auad et al. [9]. During the Delphi rounds, suggestions and observations collected from the panel of experts were considered and incorporated. The final version of the checklist had a total of 39 items, divided into nine sections. The sections included vehicle structures and adjacent areas; equipment and kitchenware; hygiene and cleanliness; food and water storage; food and water preparation and handling; residue handling; food handlers; pest and vector control; and documentation.

A pilot study was performed in five FTs. The data from the pilot study were not included in the further analysis of the study. After confirming that all the protocols elaborated for the research met their need, the data collection began. The application of the final version (39-item) of the validated evaluation instrument was conducted in 44 FTs by two trained researchers simultaneously through on-site observation to measure interrater reliability. The two researchers were instructed not to communicate with each other during the evaluation process. Each vehicle was visited once during the study. Food handlers were unaware of the date of the visit, and the application of the checklist was carried out while the food handlers were doing their routine activities. 

All items were based on adequate/inadequate/not applied answers by comparing to national food safety regulations. It is important to point out that the answer “not applied” was later translated to “adequate” or “inadequate”, depending on its context, for the correct interpretation of the data. For example, item 6.3 of the instrument refers to exclusion of food handlers with cutaneous lesions or symptoms of diseases from food handling operations. Whenever food handlers of the FT had not experienced any of those situations, the ‘not applied’ option was converted into ‘adequate’ because food handlers would not represent a source of contamination in this specific situation.

Following the application of the evaluation instrument, ready-to-eat (RTE) food samples (*n* = 44) were collected from the 44 FTs. Selection criteria for the food sample selection included the most popular dish/product from the FT according to data reported by the FT owner or manager. For this study, FTs were divided into three groups according to their RTE foods (Table 1).

All food samples were placed in a sterile plastic bag and immediately transported to the laboratory in containers with ice. Food samples were homogenized in a Stomacher (10^-1^ dilution) for 5 minutes at room temperature and then submitted to decimal serial dilutions to perform the microbiological analysis. Petrifilm™ plates (3M, St. Paul, MN, USA) were used to determine total coliforms (TC) and *Staphylococcus aureus* (SA), according to the manufacturer’s instructions. All analyses were performed in triplicate and on the same day of collection.

Considering the microbiological criteria recommended by the International Commission on Microbiological Specifications for Foods [16], food samples were considered microbiologically acceptable if coliform counts were less than 10^2^ per g or mL, while *Staphylococcus aureus* counts between 10^2^ and less than 10^3^ CFU/g were considered acceptable. These parameters were used to evaluate the food samples and to classify the FTs into contaminated and not contaminated. 

### 2.2. Stage 2—Final Version of The Evaluation Instrument

The selection of items for the final version of the evaluation instrument was conducted using two relevance criteria - statistical and practical. Using a logistic regression model, the 39 items of the instrument were ordered according to their statistical significance. Statistical relevance was also measured by the degree of association of the item’s response - adequate or inadequate—to the food sample classification of the FT—contaminated or not contaminated—using the chi-square test of independence. 

The practical relevance of items was discussed by the experts to achieve consensus. Furthermore, another Delphi round with a smaller group of four experts was conducted aiming to decide which of the non-statistically relevant items should be kept in the evaluation instrument. Items directly related to time and temperature control of food exposure, and unhygienic surfaces in direct contact with prepared food, including the hands of food handlers, were maintained. Similarly, items related to the failure of the adoption of adequate hygiene procedures for raw served foods were retained in the instrument.

After being submitted to these criteria, the evaluation instrument was reduced to a 22-item final version, of which two were classificatory items (items 8.1 and 8.2). The classificatory items were not used to perform the statistical analysis. The final instrument was used to establish an inadequacy score for the classification of FTs. The instrument score was obtained by assigning one point to each non-conforming item observed. Therefore, the instrument inadequacy score ranges from 0 to 20 points, meaning that the higher the FT score, the more inadequate it is, and, also, the higher the probability of food contamination and the higher the risk of FBD.

Risk was defined as a measure of the probability and consequence of uncertain future events and undesirable outcomes [17]. Considering that the purpose of this instrument was to assess the hygienic–sanitary conditions and practices in FTs, ‘consequence’ in this context was understood as “the occurrence of an outbreak of a FBD” and ‘probability’ was defined as “the chance of a given event happening”—which is, in this case, the contamination-, as addressed by Da Cunha et al. [5]., i.e., the contamination.

The final version of the evaluation instrument and its classification score can be found in the Supplementary Materials (Supplementary file S1).

### 2.3. Stage 3—Data Analysis

Descriptive statistics are presented as means and standard deviations for the quantitative variables and as frequencies and percentages for the categorical variables. All hypothesis tests were bicaudal with a 5% significance value. The reproducibility of the total instrument score was verified using the Intraclass Correlation Coefficient (ICC). The internal consistency of the instrument was verified by the Kuder–Richardson Formula 20 (KR-20) and Cronbach’s alpha coefficient and their discriminant validity by the area under the ROC curve (AUC). The frequency of contamination was compared between groups of FTs using the Pearson Chi-square test, and a possible difference between the instrument scores between these groups was verified by the non-parametric Mann–Whitney test, followed by the Müller–Dunn posthoc test. All statistical analyses were performed using the IBM SPSS version 22.0 software (SPSS Inc., Chicago, IL, USA).

## 3. Results

The frequency of contaminated FTs was compared between the different groups of FTs, as shown in Table 2. Among the 44 FTs that took part in this study, FTs from Group A (hot and cold sandwiches) presented the highest percentage of contamination. The lowest percentage of contamination was observed in Group C (regional and international cuisine). The Group A contamination percentage was significantly higher than that of Groups B (pizza and pasta) and C (*p* < 0.05). The percentage of contamination did not significantly differ between Groups B and C (*p* > 0.05). 

Following the application of the evaluation instrument, the inadequacy score of each FT was obtained. Considering all FTs assessed (*n* = 44), the minimum instrument score was 3, and the maximum was 18. The mean score and standard deviation of the evaluation instrument of each group is presented in Table 2. The Group A inadequacy score was significantly higher when compared to Groups B and C scores (*p* < 0.05). The scores did not significantly differ between Groups B and C. In summary, FTs of Group A also have the highest inadequacy score, meaning that this group has a higher probability of contamination than the others.

The reproducibility of the instrument was verified using the Intraclass Correlation Coefficient (ICC) considering the answers of the two evaluators that applied the instrument in the 44 FTs simultaneously and independently. ICC values range from 0 to 1, with values near to 0 meaning no agreement between the raters’ scores and values near to 1 indicating high agreement between raters’ scores. Generally, ICC values between 0.75 and 0.9 indicate good similarity [18]. The 20-item version of the instrument obtained an ICC value of 0.780 (CI 95%: 0.597; 0.880), indicating good reproducibility of the instrument.

The Kuder–Richardson Formula 20 (KR-20) and the Cronbach’s alpha coefficient were used for the determination of internal consistency. KR-20 and Cronbach’s alpha values range from 0 to 1, with values near to 0 meaning no reliability and values near to 1 indicating perfect reliability. KR-20 values above 0.5 are considered reasonable [19], while a Cronbach’s alpha coefficient above 0.6 is considered satisfactory [20]. The final version of the instrument presented a KR-20 value of 0.627 and a Cronbach’s alpha coefficient of 0.634, indicating good internal consistency.

The evaluation instrument score was used to perform the classification of FTs, which was defined by the levels of sensitivity and specificity. Considering the contamination as the outcome of interest, sensitivity represents the probability of the instrument to present a positive result in contaminated FTs, while specificity represents the probability that the instrument will present a negative result in noncontaminated FTs. As shown in Table 3, the cutoff point 12 represents the maximization of both sensitivity (83.3%) and specificity (61.5%) (area under the ROC curve = 0.723; CI 95%: 0.571; 0.875).

This cutoff point can be adopted when the two possible classification errors have the same severity. In this case, a FT would be considered at risk of contamination if it presented at least 12 inadequate items out of the 20 evaluated. As shown in Table 4, when exhibiting a score of up to 11 nonconforming points, the probability of contamination is approximately 19%, meaning a low risk of contamination. Contrarily, if a FT displays a score of 12 points or higher, the probability of contamination is 60.9%, meaning a high risk of contamination. 

Table 5 lists the items of the final version of the instrument, along with the frequency of responses assigned to the 44 FTs assessed. 

## 4. Discussion

This study is considered a continuation of our previous research [9], in which we developed a checklist for the assessment of the hygienic–sanitary practices and conditions in FTs and established its content validity by the Delphi technique. This technique allows the implementation of an expert panel in order to perform the content validation, facilitating the achievement of consensus on the experts’ opinions [21]. The use of the Delphi method allows for the ability to have a great volume of information; better reflection on the subject and more elaborated answers due to the use of questionnaires; elimination of influences of judgment that could interfere with the quality of the answers due to the anonymity of the technique; and the possibility of incorporating new ideas raised by experts in the area [22]. However, it is important to evaluate if the construction and applicability allow the true measurement of what one intends to measure, under real circumstances for which the instrument was designed to. Therefore, in the current study, we explored the construct validity and the response process as sources of evidence for validity.

According to Messick [23], validity “always refers to the degree to which empirical evidence and theoretical rationales support the adequacy and appropriateness of interpretations and actions based on test scores”. Therefore, validity is a crucial point concerning the process of questionnaire construction, as it aims to demonstrate the extent to which the scores from a measure represent the variable they are intended to. Validity also allows one to make inferences and correct interpretations of the results when applying a questionnaire, as well as to establish a relation with the construct/variable measured [24].

Validity requires the accumulation of theoretical and empirical support for the appropriateness of the claims [25]. The theoretical rationales required to achieve the validity argument in our study were demonstrated by content validity, performed in our previous study, and construct validity, demonstrated in this study. The construct validity concerns the simultaneous process of measure and theory validation [26], and is defined as the degree to which an instrument measures the trait or theoretical construct that it is intended to measure [27]. The development of the evaluation instrument was guided by the structured model of risk assessment and theoretically founded by the conceptual framework of risk and hazard variables, which were used to build the foodborne disease empirical construct. The empirical support to provide a validity argument was achieved by the evidence based on the response process, which consisted of the application of the previously validated instrument under real conditions, as well as its psychometric analyses and test score interpretation and use. 

The application of the validated evaluation instrument to 44 FTs in the Federal District revealed that none achieved 100% adequacy. A high level of inadequacy is directly correlated with poor adherence to good manufacturing practices and conditions and, consequently, to a higher probability of food contamination and higher risk of FBD. The higher inadequacy percentages were identified in categories including ‘vehicle structure and adjacent areas’, ‘equipment and kitchenware’, ‘residue handling’ and ‘food handlers’. As a particular kind of street food service, FTs share the same main issues concerning street food. Previous studies have demonstrated that the street food sector is fraught with unwholesome activities that may pose concern over the safety of practitioners and, especially, the health of consumers [3,28,29]. Despite sharing several common features, the FT industries worldwide have particularities, including climate conditions and the main factors that can cause food contamination and outbreaks. The instrument assessment described in this study is considered relevant and applicable to the Brazilian context and, therefore, further studies should consider its adaptation to local reality.

Concerning the classification of the FTs, a higher inadequacy percentage was found in Group A (hot and cold sandwiches), which also presented the highest rate of contaminated samples. Despite being considered traditional meals, the sandwiches and hot dogs served by the FTs assessed had a modern/sophisticated touch, including gourmet ingredients and special gravies and sauces, which consequently implies a high number of ingredients and food preparation processes. Thus, the contamination results of Group A could be due to improper conditions or contamination throughout the sandwich production chain, including inadequate storage temperature or insufficient cooking of the meat. Chilled ingredients should be kept in temperatures that might not result in risk to health, and, when applicable, should be thoroughly cooked (including the geometric center in the case of meats) to ensure significant reduction of eventual microorganism counts [30], which may not have occurred. Contamination results of Group A could also be due to the absence or inadequate sanitation of the raw vegetables or leaves. Some studies have demonstrated that biological contamination of fresh leafy and ready-to-eat vegetables are a serious public health problem worldwide, including in Brazil, since various factors must be considered to guarantee the effectiveness of their sanitation [31,32,33]. On the other hand, a possible explanation for the low rate of contamination observed in Groups B and C may be the fact that these foods are subjected to high temperatures, especially sauces, which are subjected to boiling temperatures that can minimize the probability of food contamination.

Considering the inadequacy score obtained in the application of the evaluation instrument, a cutoff point was proposed for the classification of contamination probabilities of FTs. The cutoff-set at 11 points was defined in order to minimize the probability of a false negative (to classify as adequate a FT at probability of contamination), meaning that if a FT scores up to 11 points in the inadequacy score, its probability of providing contaminated food (probability of a false negative) is 15.8%. The adopted cutoff point correctly classified 77.8% (sensitivity of 77.8%) of the contaminated FTs and correctly classified 65.4% of the non-contaminated FTs (specificity of 65.4%). 

The psychometric analyses were performed to expand the reliability of the evaluation of the hygienic–sanitary practices and conditions assessed by this instrument. The reproducibility of the checklist was verified using the Intraclass Correlation Coefficient (ICC) and achieved a value of 0.780 (CI 95%: 0.597; 0.880), which indicates good reproducibility of the instrument. Additionally, the analysis of internal consistency using the Kuder–Richardson Formula 20 (KR-20) and the Cronbach’s alpha presented values of 0.627 and 0.634, indicating good internal consistency. Considering that reliability, along with validity metrics, were performed in this study, the evaluation instrument can be considered a reliable and evidence-based assessment tool.

The adoption of valid and standardized instruments that are also brief, easily accessible and practical can effectively assist in the decision-making process. In the food safety area, it can also provide rapid and accurate identification of the hazards present and, consequently, the development of targeted intervention strategies for the prevention and control of foodborne diseases. Long and complex instruments may be time-consuming and complicated to complete, leading to exhaustion. Therefore, the shorter version of the evaluation instrument presented in this study is desirable because it captured the most important information in a small number of items sufficient to represent the key elements. Additionally, it can also represent a more cost-effective alternative to microbiological sampling, considering that it can act as an early warning, with the risks being identified and controlled before pathogens contaminate the food. The simple interpretation of the instrument score is another positive feature of the evaluation instrument that may encourage its application. However, the extended version may serve as guidance for FTs willing to comprehend and implement proper food safety practices. The findings of this study revealed that the Brazilian FT sector still faces food safety challenges. However, these issues may be addressed by the supervision and guidance from the application of the evaluation instrument of this study, providing consumer access to safe food and reducing the risk of FBD outbreaks.

## 5. Conclusions

An evaluation instrument for the hygienic–sanitary conditions of food trucks was applied to food truck in the Federal District. The results of this study provide a shorter version of the original checklist proposed by Auad et al. [9], with a scoring system, which represents a useful and objective tool in managing food safety risks and assisting in real-time decisions for the prevention of FBD in food trucks. Additionally, the good reproducibility and internal consistency of the evaluation instrument indicate its reliability.

The availability of a published description of the development and validation of a scientifically founded and risk-based tool, as well as the results of its application, may assist in the adoption of strategies for the prevention of FBD outbreaks in the food truck sector and, thus, for the protection of consumers’ health.

## Figures and Tables

**Table 1:** Classification of food trucks according to their ready-to-eat (RTE) food groups.

Ready-to-Eat Food Groups	Description	Number of Samples
**Group A**	Hot and Cold Sandwiches (e.g., hamburgers, hot dogs)	20
**Group B**	Pizza and Pasta (e.g., pizza, spaghetti, risotto, *crepes*)	13
**Group C**	Regional and international cuisine (e.g., Arabian cuisine, Japanese cuisine, Bahian cuisine)	11

**Table 2:** Frequency and percentage of contaminated food trucks and mean score and standard deviation of the evaluation instrument, according to food truck group classification.

Group	A	B	C	*p*-Value
**Contamination (*n*, %)**				
Non-contaminated	7 (35%)	10 (76.9%)	9 (81.8%)	0.014 *
Contaminated	13 (65%) ^A^	3 (23.1%) ^B^	2 (18.2%) ^B^	
**Score (mean; SD)**	13.05 (2.70) ^A^	9.85 (3.11) ^B^	10.27 (2.33) ^B^	0.004 **

* Chi-square test with Monte Carlo approximation; ** Kruskal–Wallis test with Müller–Dunn posthoc test. ^A^^, B^ Groups with the same letters do not differ significantly.

**Table 3:** Probability of contamination of food truck defined by sensitivity and specificity levels according to the cutoff point adopted for the evaluation instrument.

Contaminated Food Truck if the Score Is Equal or Higher than	Sensitivity	Specificity
3	1.000	0.000
5	1.000	0.077
7	1.000	0.115
8	0.944	0.154
9	0.944	0.192
10 ^1^	0.944	0.308
11	0.889	0.462
12 ^2^	0.778	0.654
13	0.389	0.731
14	0.278	0.885
15	0.222	0.962
16	0.222	1.000
17	0.167	1.000
18	0.111	1.000
19	0.000	1.000

^1^ Cutoff point which sets sensibility in 94.4%; ^2^ Cutoff point which maximizes both sensitivity and specificity.

**Table 4:** Classification of food trucks considering the cutoff point 12.

Score	Contaminated	Non-Contaminated	Total
Up to 11	4 (19.0%)	17 (81.0%)	21
12 or more	14 (60.9%)	9 (39.1%)	23
Total	18	26	44

**Table 5:** Items and frequency responses of the final version of the evaluation instrument applied to the food trucks assessed (*n* = 44).

Evaluation Instrument Items	Adequate (*n*, %)	Inadequate (*n*, %)
*1. Vehicle structure and adjacent areas*		
1.1 Internal surfaces (walls, floor and ceiling) are properly conserved, free of cracks, drips, molds and dehulling. They are designed to facilitate maintenance, cleaning and, when appropriate, disinfection operations (i.e., no hard-to-reach quarters and areas, without equipment located in order to prevent or hinder cleaning).	3 (6.8%)	41 (93.2%)
1.2 Surfaces in direct contact with food are smooth, resistant, waterproof and easily sanitized (stainless steel, mineral solid surface and similar). They are properly conserved, free of dirt, cracking and peeling.	18 (40.9%)	26 (59.1%)
1.3 Surfaces in direct contact with food are designed to facilitate maintenance, cleaning and disinfection operations, with no hard-to-reach quarters and areas, with no equipment located to prevent or hinder cleaning.	36 (81.8%)	8 (18.2%)
1.4 The water tank is of smooth, waterproof and resistant material (polyethylene, polypropylene, stainless steel and similar), cleaned, rust free, suitable for carrying out activities (hygiene of hands, utensils, equipment, and surfaces), kept capped and stocked with drinking water.	32 (72.7%)	12 (27.3%)
*2. Equipment and kitchenware*		
2.1 Equipment and utensils are clean and properly conserved. They allow proper cleaning, are of non-toxic material and, if necessary, are heat-resistant and with a protection and safety device.	14 (31.8%)	30 (68.2%)
2.2 Equipment and utensils used at all stages of food preparation are stored and are washed after use and sanitized before use or are disposable. They are protected from dust and contaminants when stored.	4 (9.1%)	40 (90.9%)
*3. Food and water storage*		
3.1 Ingredients, raw material and pre-prepared and ready-to-eat foods are stored off the floor, in a clean space and separately away from disposable and cleaning materials.	26 (59.1%)	18 (40.9%)
3.2 Perishable ingredients, raw material, and pre-prepared and ready-to-eat foods are stored separately according to food groups (milk and dairy products, meat, vegetables) and categories (raw and cooked, sanitized and unsanitized), under refrigeration or freezing temperature.	13 (29.5%)	31 (70.5%)
3.3 Semi perishable ingredients, raw material and pre-prepared and ready-to-eat foods are stored and separated according to food groups (cereals, legumes, sugars, oils) and preserved as instructed by the manufacturer or supplier and/or are packed in containers with a lid, waterproof, washable and non-toxic material or plastic bags suitable for food.	24 (54.5%)	20 (45.5%)
*4. Food and water preparation and handling*		
4.1 There are no crosses, i.e. there is no direct or indirect contact between raw and cooked food or between sanitized and unsanitized food at all stages of food preparation (pre-preparation, preparation, distribution).	29 (65.9%)	15 (34.1%)
4.2 Perishable ingredients, raw material and pre-prepared and ready-to-eat foods are stored in the refrigerator or freezer. They are exposed to room temperature only for the minimum time required for handling, with maximum preparation time at room temperature of 30 min.	10 (22.7%)	34 (77.3%)
4.3 Ingredients, raw material and food submitted to cooking reach temperature and time of 65 °C/15 min, 70 °C/2 min, 74 °C in the geometric center or other combinations of the binomial time/temperature that ensure hygienic-sanitary quality.	33 (75.0%)	11 (25.0%)
4.4 Fruits, vegetables and legumes, if not previously sanitized, are submitted to sanitation, using products regularized by ANVISA or Ministry of Health, and obeying the manufacturer’s instructions.	24 (54.5%)	20 (45.5%)
*5. Residue handling*		
5.1 Waste collectors in the internal and external areas of the vehicle are clean, kept capped and covered with bags suitable for the activity. They are hands-free lids (with pedal, sensor or similar) and are emptied before reaching their maximum capacity and bags are discarded in an appropriate place.	8 (18.2%)	36 (81.8%)
*6. Food handlers*		
6.1 The personal hygiene routine of handlers includes the use of preserved and clean uniforms that are used exclusively during food handling operations, with protection against direct contact with food (coat or apron).	1 (2.3%)	43 (97.7%)
6.2 The personal hygiene routine of manipulators includes the use of hair trapped and protected with a cap and the use of a coat or apron. In case of a beard and mustache, a mask should be used. Nails are clean, short, with no enamel or base. No wearing of adornments (necklaces, amulets, bracelets, ribbons, earrings, nails and false eyelashes, piercing on exposed areas, watches, rings and rings) during food handling operations.	8 (18.2%)	36 (81.8%)
6.3 Handlers with cutaneous lesions and wounds or symptoms of diseases/infections (i.e. respiratory, gastrointestinal, ocular) are excluded from food handling operations.	31 (70.5%)	13 (29.5%)
6.4 Handlers do not smoke, sing, whistle, sneeze, spit, cough, eat, handle money or practice other acts that may contaminate food during food handling operations.	13 (29.5%)	31 (70.5%)
6.5 Handlers carefully wash their hands when they arrive at work, before and after handling the food, after any interruption of service, after touching contaminated materials, after using the toilets and whenever necessary. Under the impossibility of washing hands, handlers wear disposable gloves in place of utensils to handle only ready-to-eat foods and previously sanitized fruits and vegetables, replacing them and disposing of them as soon as they discontinue the procedure and before touching another food or surface that is not part of the preparation.	22 (50.0%)	22 (50.0%)
*7. Pest and vector control*		
7.1 There is a set of effective and continuous control actions to prevent the attraction, shelter, access and proliferation of vectors and pests in the internal area of the vehicle.	29 (65.9%)	15 (34.1%)
*8. Documentation*		
8.1 ^1^ Existence of a food handler proven to be able to implement and supervise Good Practices.	28 (63.6%)	16 (36.4%)
8.2 ^1^ Existence of a Manual of Good Practices and Standard Operating Procedures (SOP) of the whole process (reception of raw materials, storage, pre-preparation, preparation and distribution, hygiene and health, hygiene of facilities, equipment and utensils; water reservoir, waste control, vector control and pest control).	24 (54.5%)	20 (45.5%)

^1^ Classificatory item.

## Supplementary Materials

The following are available online at http://www.mdpi.com/2072-6643/11/2/430/s1, File S1: Checklist for the hygienic–sanitary diagnostic of food trucks.
