# Supplementary material for: Food Trucks: Assessment of an Evaluation Instrument Designed for the Prevention of Foodborne Diseases
Source: Nutrients. 2019 Feb 19;11(2):430. doi: 10.3390/nu11020430 (PMC6412621; doi:10.3390/nu11020430)
Supplement: Supplementary file 1 [file nutrients-11-00430-s001.pdf]

File S1: CHECKLIST FOR THE HYGIENIC-SANITARY DIAGNOSTIC OF FOOD TRUCKS

Legend

A - Adequate I - Inadequate NA - Not Applicable OBS - Observation

**Number:**	**Year:**
**Food Truck:**	
**Owner:**	
**Health License:**	
**Email/Phone:**	
**City/State:**	**Zip Code:**

**1. Vehicle Structure and Adjacent Areas**	**A**	**I**	**NA**	**OBS**
1.1 Internal surfaces (walls, floor and ceiling) are properly conserved, free of cracks, drips, molds and dehulling. They are designed to facilitate maintenance, cleaning and, when appropriate, disinfection operations (i.e. no hard-to-reach quarters and areas, without equipment located in order to prevent or hinder cleaning).				
1.2 Surfaces in direct contact with food are smooth, resistant, waterproof and easily sanitized (stainless steel, mineral solid surface and similar). They are properly conserved, free of dirt, cracking and peeling.				
1.3 Surfaces in direct contact with food are designed to facilitate maintenance, cleaning and disinfection operations, with no hard-to-reach quarters and areas, with no equipment located to prevent or hinder cleaning.				
1.4 The water tank is of smooth, waterproof and resistant material (polyethylene, polypropylene, stainless steel and similar), cleaned, rust free, suitable for carrying out activities (hygiene of hands, utensils, equipment, and surfaces), kept capped and stocked with drinking water.				
**2. Equipment and Kitchenware**	**A**	**I**	**NA**	**OBS**
2.1 Equipment and utensils are clean and properly conserved. They allow proper cleaning, are of non-toxic material and, if necessary, are heat-resistant and with a protection and safety device.				
2.2 Equipment and utensils used at all stages of food preparation are stored and are washed after use and sanitized before use or are disposable. They are protected from dust and contaminants when stored.				
**3. Food and Water Storage**	**A**	**I**	**NA**	**OBS**
3.1 Ingredients, raw material and pre-prepared and ready-to-eat foods are stored off the floor, in a clean space and separately away from disposable and cleaning materials.				
3.2 Perishable ingredients, raw material, and pre-prepared and ready-to-eat foods are stored separately according to food groups (milk and dairy products, meat, vegetables) and categories (raw and cooked, sanitized and unsanitized), under refrigeration or freezing temperature.				
3.3 Semi perishable ingredients, raw material and pre-prepared and ready-to-eat foods are stored and separated according to food groups (cereals, legumes, sugars, oils) and preserved as instructed by the manufacturer or supplier and/or are packed in				

	containers with a lid, waterproof, washable and non-toxic material or plastic bags suitable for food.				
**4.**	**Food and Water Preparation and Handling**	**A**	**I**	**NA**	**OBS**
4.1	There are no crosses, i.e. there is no direct or indirect contact between raw and cooked food or between sanitized and unsanitized food at all stages of food preparation (pre-preparation, preparation, distribution).				
4.2	Perishable ingredients, raw material and pre-prepared and ready-to-eat foods are stored in the refrigerator or freezer. They are exposed to room temperature only for the minimum time required for handling, with maximum preparation time at room temperature of 30 minutes.				
4.3	Ingredients, raw material and food submitted to cooking reach temperature and time of 65ºC/15min, 70ºC/2min, 74ºC in the geometric center or other combinations of the binomial time/temperature that ensure hygienic-sanitary quality.				
4.4	Fruits, vegetables and legumes, if not previously sanitized, are submitted to sanitation, using products regularized by ANVISA or Ministry of Health, and obeying the manufacturer`s instructions.				
4.5	Oils and fats used for frying have their quality guaranteed by means of temperature control (heated to a maximum of 180ºC), observation of sensorial characteristics (no change in color, odor and taste and no presence of smoke and foam) and/or use of a pH indicator strip.				
**5.**	**Residue Handling**	**A**	**I**	**NA**	**OBS**
5.1	Waste collectors in the internal and external areas of the vehicle are clean, kept capped and covered with bags suitable for the activity. They are hands-free lids (with pedal, sensor or similar) and are emptied before reaching their maximum capacity and bags are discarded in an appropriate place.				
**6.**	**Food Handlers**	**A**	**I**	**NA**	**OBS**
6.1	The personal hygiene routine of handlers includes the use of preserved and clean uniforms that are used exclusively during food handling operations, with protection against direct contact with food (coat or apron).				
6.2	The personal hygiene routine of manipulators includes the use of hair trapped and protected with a cap and the use of a coat or apron. In case of a beard and mustache, a mask should be used. Nails are clean, short, with no enamel or base. No wearing of adornments (necklaces, amulets, bracelets, ribbons, earrings, nails and false eyelashes, piercing on exposed areas, watches, rings and rings) during food handling operations.				
6.3	Handlers with cutaneous lesions and wounds or symptoms of diseases/infections (i.e. respiratory, gastrointestinal, ocular) are excluded from food handling operations.				
6.4	Handlers do not smoke, sing, whistle, sneeze, spit, cough, eat, handle money or practice other acts that may contaminate food during food handling operations.				
6.5	Handlers carefully wash their hands when they arrive at work, before and after handling the food, after any interruption of service, after touching contaminated materials, after using the toilets and whenever necessary. Under the impossibility				

		A	I	NA	OBS
	of washing hands, handlers wear disposable gloves in place of utensils to handle only ready-to-eat foods and previously sanitized fruits and vegetables, replacing them and disposing of them as soon as they discontinue the procedure and before touching another food or surface that is not part of the preparation.				
**7.**	**Pest and Vector Control**	**A**	**I**	**NA**	**OBS**
7.1	There is a set of effective and continuous control actions to prevent the attraction, shelter, access and proliferation of vectors and pests in the internal area of the vehicle.				
**8.**	**Documentation**	**A**	**I**	**NA**	**OBS**
8.1	Existence of a food handler proven to be able to implement and supervise Good Practices.				
8.2	Existence of a Manual of Good Practices and Standard Operating Procedures (SOP) of the whole process (reception of raw materials, storage, pre-preparation, preparation and distribution, hygiene and health, hygiene of facilities, equipment and utensils; water reservoir, waste control, vector control and pest control).				
	**Final Score**				

**Final Score Instructions**	
. For each 'inadequate' item, assign one point to the final score.<br>. For 'adequate' items, do not assign any points to the final score.<br>. 'Not applicable' items must be converted to 'adequate' or 'inadequate' according to the context (whether the item represents or not a source of contamination risk) for the correct interpretation of the results.	
**Classification**	
Food trucks with ≤ 11 inadequate items: low risk of contamination	
Food trucks with ≥ 12 inadequate items: high risk of contamination	
